# SG-APSIC1062: The role of active surveillance in the primary-care setting during a pandemic in Singapore

**DOI:** 10.1017/ash.2023.23

**Published:** 2023-03-16

**Authors:** Chau Chain Yan, Giselle Li, Lim Min Wei

**Affiliations:** Singapore National University Polyclinics, Singapore; Singapore National University Polyclinics, Singapore; Singapore National University Polyclinics, Singapore

## Abstract

**Objectives:** In response to the COVID-19 pandemic, primary care swiftly transformed and re-established patient flow in clinics to red, orange, and green zones based on a set of screening criteria. To further manage the influx of suspected COVID-19 patients and their needs safely, a list of surveillance audit criteria was developed to ensure good infection control standards. **Methods:** The infection control team prepared the surveillance audit criteria based on recommended CDC/WHO guidelines for pandemic preparedness. These criteria were contextualized to the primary-care polyclinic setting. The surveillance audit criteria were grouped according to their category: screening, triage, early recognition and source control, inventory management of personal protective equipment (PPE), infection control measures in the red zone, precautionary measures during collection of nasopharyngeal swabs and environmental cleaning and disinfection for premises in the red, orange, and green zones, respectively. The infection control liaison nurses in each polyclinic were trained to use the checklist to ensure consistency in interpretation of the criteria. **Results:** Surveillance audits were conducted biweekly in the first 3 months then monthly once the compliance rate was steady at 90%–100% for all categories. The overall average compliance rate since commencing in March 2020 for all polyclinics was sustained at 90%–100%. Common findings included inappropriate use of PPE (eg, self-contamination during removal of gown or wrong sequence of doffing), inadequate ventilation, and inadequate cleaning processes. All findings were corrected immediately, and staff education was provided. **Conclusions:** Primary care plays an important role during a pandemic. It is essential that both patients and healthcare workers in the primary care setting are protected from infection risk during a pandemic. Having a good surveillance audit process helps ensure that primary care services can continue for the general population. Surveillance is an essential component of the health system’s response to a pandemic.

